# Non-Alcoholic Fatty Liver Disease Is an Independent Risk Factor for LDL Cholesterol Target Level

**DOI:** 10.3390/ijerph18073442

**Published:** 2021-03-26

**Authors:** Jun-Hyuk Lee, Hye Sun Lee, A-Ra Cho, Yong-Jae Lee, Yu-Jin Kwon

**Affiliations:** 1 Department of Family Medicine, Nowon Eulji Medical Center, Eulji University, Seoul 01830, Korea; swpapa@eulji.ac.kr; 2 School of Medicine, Eulji University, Daejeon 34824, Korea; 3 Biostatistics Collaboration Unit, Department of Research Affairs, Yonsei University College of Medicine, Seoul 06273, Korea; HSLEE1@yuhs.ac; 4 Department of Family Medicine, Yonsei University College of Medicine, Gangnam Severance Hospital, Seoul 06273, Korea; ara1713@yuhs.ac (A.-R.C.); ukyjhome@yuhs.ac (Y.-J.L.); 5 Department of Family Medicine, Yonsei University College of Medicine, Yongin Severance Hospital, Yongin-si 16995, Korea

**Keywords:** non-alcoholic fatty liver disease, low-density lipoprotein cholesterol, cardiovascular disease

## Abstract

Although patients with non-alcoholic fatty liver disease (NAFLD) face a higher risk of cardiovascular disease (CVD), it is not known whether people with NAFLD are less likely to achieve optimal management of low-density lipoprotein (LDL) cholesterol than those without NAFLD. We aimed to investigate the longitudinal effect of NAFLD on the management of LDL cholesterol in 5610 adults from the Korean Genome and Epidemiology Study. Participants were classified into NAFLD and normal groups. Non-achievement of the target LDL cholesterol level was set according to one’s cardiovascular disease (CVD) risk level. The estimated proportion of individuals who did not achieve their LDL cholesterol targets was higher in the NAFLD group than in the normal group during the follow-up period of 12 years in a generalized estimation equation model. Multivariable Cox regression analysis revealed a hazard ratio and 95% confidence interval for incident non-achievement of one’s LDL cholesterol target of 1.196 (1.057–1.353) in the NAFLD group (*p* = 0.005). We found that NAFLD was significantly related to non-achievement of LDL cholesterol targets in this prospective cohort study. Prevention and proper management of NAFLD have important health implications for the prevention of CVD.

## 1. Introduction

Cardiovascular disease (CVD) is a major cause of morbidity and mortality worldwide [1]. According to the 2017 World Health Organization fact sheet, approximately 31% of all deaths (17.9 million people) stemmed from CVD in 2016 [2]. Risk factors for CVD can be classified into non-modifiable and modifiable risk factors. Non-modifiable risk factors include age, sex, ethnicity, and family history of premature coronary artery disease; modifiable risk factors include smoking, diabetes, high blood pressure, obesity, physical inactivity, and high blood cholesterol. Individuals who are at high risk or have already experienced CVD are recommended to manage modifiable CVD risk factors, including behavioral factors [3,4,5]. Among the modifiable metabolic risk factors, low-density lipoprotein (LDL) cholesterol is a well-known risk factor for the development of atherosclerosis [6]. To reduce CVD risk as much as possible, current guidelines recommend optimizing LDL cholesterol management based on an individual’s CVD risk level [3,6,7].

The liver plays a major role in lipid metabolism [8]. Non-alcoholic fatty liver disease (NAFLD), including non-alcoholic steatohepatitis and liver cirrhosis, has become the most common cause of chronic liver disease worldwide [9]. NAFLD is usually accompanied by metabolic disorders, such as type 2 diabetes, obesity, and dyslipidemia [10]. Therefore, the risk of CVD among NAFLD patients is likely to be higher than it is in normal individuals. Interestingly, a longitudinal follow-up study showed that the most common cause of death in NAFLD patients is cardiovascular disease (CVD), not liver-related disease [11]. Thus, a multidisciplinary patient-centered and personalized medicine approach might be needed to effectively prevent CVD in NAFLD patients. However, there is no evidence on whether people with NAFLD are less likely to achieve optimal management of LDL cholesterol than people without NAFLD.

Therefore, in this study, we aimed to investigate the effect of NAFLD on the management of LDL cholesterol based on one’s CVD risk.

## 2. Materials and Methods

### 2.1. Study Population

We used data from the Korean Genome and Epidemiology Study (KoGES) (known as the KoGES_Ansan and Ansung study). The KoGES_Ansan and Ansung study is a longitudinal prospective cohort study initiated by the Korean National Institute of Health to verify risk factors for non-communicable diseases [12]. A total of 10030 community-dwellers aged 40–69 years in urban (Ansan) and rural (Ansung) areas were recruited in the baseline survey (2001–2002). The survey was conducted biennially up to 2013–2014 (sixth follow-up).

**Figure 1:**
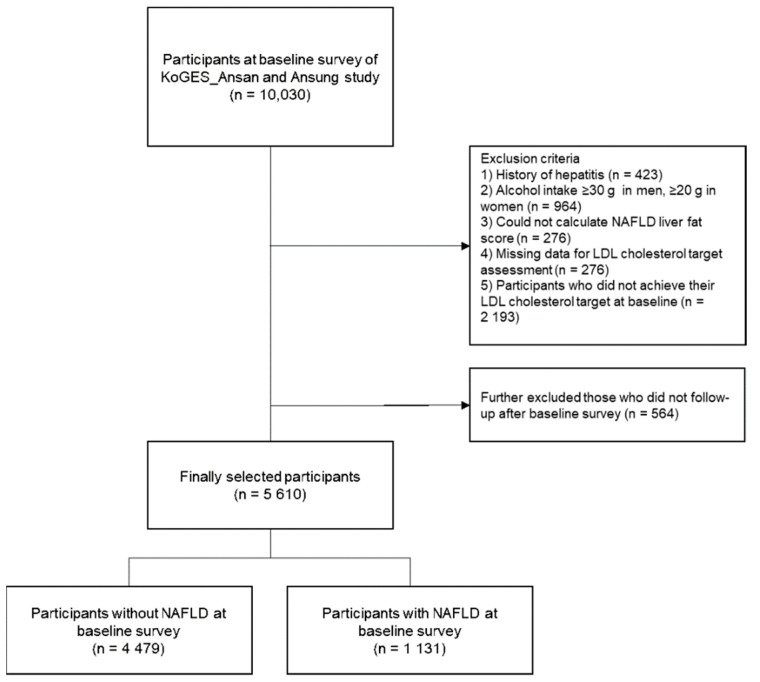
Flowchart of study population selection. Abbreviations: Korean Genome and Epidemiology Study (KoGES); low-density lipoprotein (LDL); non-alcoholic fatty liver disease (NAFLD).

From the 10030 participants in the baseline survey, we excluded those who (1) had a history of hepatitis (*n* = 423), (2) consumed ≥30 g of alcohol per day in men or ≥20 g per day in women (*n* = 964), (3) had no data with which to calculate NAFLD liver fat scores (*n* = 276), (4) had no data with which to evaluate LDL cholesterol targets according to individual CVD risk (*n* = 276), and (5) achieved their LDL cholesterol target at the baseline survey (*n* = 2193). Of the remaining 6174 participants, 564 participants who had no follow-up data after the baseline survey were further excluded. Finally, a total of 5610 participants (1131 participants with NALFD and 4479 participants without NAFLD) were analyzed in the study (Figure 1). Informed consent was obtained from all eligible participants. This study was approved by the Institutional Review Board (IRB) of Yongin Severance Hospital (IRB number: 9-2020-0043).

### 2.2. Assessment of NAFLD

NAFLD was defined using the previously validated fatty liver prediction model, the NAFLD-liver fat score, with the following formula: −2.89 + 1.18 × metabolic syndrome (Yes: 1, No: 0) + 0.45 × diabetes mellitus (Yes: 2, No: 0) + 0.15 × insulin in µIU/mL + 0.04 × AST in U/L–0.94 × aspartate aminotransferase (AST)/alanine aminotransferase (ALT). An NAFLD-liver fat score > −0.640 was considered indicative of having NALFD [13].

### 2.3. Assessment of LDL Cholesterol Target Levels According to CVD Risk

LDL cholesterol levels were calculated using the Friedewald equation for participants with serum triglyceride levels less than 400 mg/dL as follows: LDL cholesterol (mg/dL) = total cholesterol − high-density lipoprotein (HDL) cholesterol − (triglyceride/5) [14].

The major risk factors for CVD included five items: (1) men aged ≥ 45 years and women aged ≥ 55 years; (2) systolic blood pressure (SBP) ≥ 140 mmHg and diastolic blood pressure (DBP) ≥ 90 mmHg or current treatment with antihypertensive medications for more than 20 days per month; (3) current smoker; (4) HDL cholesterol level < 40 mg/dL; and (5) family history of premature CVD developing before the age of 55 years in men and before the age of 65 years in women among an individual’s parents and/or siblings. HDL cholesterol ≥ 60 mg/dL was considered as a protective factor for CVD risk [7].

We categorized the participants into four risk groups (low risk, moderate risk, high risk, and very high risk) based on their total CVD risk level. Participants who had no or one CVD risk factor (among the major five risk factors for CVD) were classified into the low-risk group. The moderate-risk group comprised participants with two or more major risk factors for CVD. Participants with diabetes mellitus without signs of target organ damage were classified into the high-risk group. The very high-risk group consisted of (1) participants who had experienced coronary artery disease, ischemic stroke, or transient ischemic attack; (2) patients with diabetes with signs of target organ damage (glomerular filtration rate < 60 mL/min/1.73 m^2^, albuminuria, concurrence of hypertension); or (3) diabetic patients who were current smokers.

The LDL cholesterol management target levels according to each individual’s CVD risk level were set as LDL cholesterol level < 160 mg/dL for the low-risk group, <130 mg/dL for the moderate-risk group, <100 mg/dL for the high-risk group, and <70 mg/dL for the very high-risk group, respectively [7].

Finally, we defined non-achievement of LDL cholesterol target as a higher LDL cholesterol level than the LDL cholesterol management targets based on one’s CVD risk.

### 2.4. Covariates

Height (m) and body weight (kg) were measured to the nearest 0.1 cm and 0.1 kg, respectively. Body mass index (BMI) was calculated as body weight divided by height squared (kg/m^2^). Waist circumference (WC) was measured in the horizontal plane midway between the lowest rib and the iliac crest (cm). Obesity was defined as a BMI ≥ 25 mg/m^2^ according to the diagnostic criterion for obesity defined by the Korean Society for the Study of Obesity [15]. SBP and DBP were defined as the average of the last two of three measured values taken at 5-min intervals. Mean blood pressure (MBP) was then calculated using the equation (SBP + 2 × DBP)/3.

All blood tests were performed after at least 8 hours of overnight fasting. Plasma glucose level, serum concentrations of total cholesterol, triglyceride, and HDL cholesterol, AST, and ALT were measured enzymatically using a Chemistry Analyzer (Hitachi 7600, Tokyo, Japan by August 2002 and ADVIA 1650, Siemens, Tarrytown, NY from September 2002). C-reactive protein (CRP) concentrations were measured by means of an immunoradiometric assay (ADVIA 1650, Siemens, Tarrytown, NY, USA). 

History of smoking status, drinking status, employment status, and monthly income were collected during an interview. A current smoker was defined as one who smokes currently and has smoked at least 100 cigarettes during his/her lifetime. We divided alcohol drinking status into two categories: currently drinking or not. We also calculated the amount of alcohol intake consumed by the participants according to the number of grams of alcohol consumed per day (g/day). We regarded one episode of exercise as exercise for at least 30 min [16]. Then, we categorized physical activity into two groups: regular exercise (more than three episodes per week) or not (less than two episodes per week). Employment status was divided into two groups: employed and unemployed. Monthly income was categorized into three groups: less than one million Korean Won, 1–2 million Korean Won, and more than 2 million Korean Won. We defined the presence of chronic diseases as having at least one of comorbid conditions such as chronic obstructive pulmonary disease or any chronic cancer, excluding other chronic diseases (diabetes mellitus, stroke, myocardial infarction, and chronic kidney disease stages 3 to 5) used to assess the LDL cholesterol management target level.

For dietary surveillance, a validated food frequency questionnaire (FFQ) consisting of 103 food items was used [17,18]. Daily total calorie intake (kcal/day), protein intake (g/day), fat intake (g/day), and carbohydrate intake (g/day) were calculated through the FFQ.

### 2.5. Statistical Analysis

All data are presented as numbers (percentages, %) for categorical variables and means ± standard deviations (SD) or medians (25th, 75th) for continuous variables. For continuous variables, the independent *t*-test or Mann–Whitney U-test were performed to compare differences between the two study groups. The Chi-squared test was used to compare categorical variables. We used a generalized estimation equation (GEE) to assess the long-term effects of NAFLD, while considering correlations between measurements over time. The cumulative incidence of non-achievement of LDL cholesterol target was represented by Kaplan–Meier curves. The log-rank test was used to determine if distributions of the cumulative incidence of non-achievement of LDL cholesterol target differed between groups. Multivariable Cox proportional hazards regression models were used to calculate hazard ratios (HRs) and 95% confidence intervals (CIs) for incident non-achievement of LDL cholesterol targets after adjusting for potential confounding variables. Subgroup analyses for age group, sex, obesity status, physical activity, smoking status, and alcohol drinking status were also performed.

All statistical analyses were conducted using SAS statistical software (version 9.4; SAS Institute Inc., Cary, NC, USA) and R (Version 4.0.3; R Foundation for Statistical Computing, Vienna, Austria). The significance level was set at *p* < 0.05.

## 3. Results

### 3.1. General Characteristics of the Study Population

The baseline characteristics of the study population are shown in Table 1. Among all 5610 participants, 1131 (20.2%) had NAFLD. The proportion of male was significantly higher in the NAFLD group than the normal group. The mean age ± SD was 51.8 ± 8.8 in the normal group and 52.9 ± 8.6 in the NAFLD group (*p* < 0.001), respectively. The mean BMI, WC, MBP, serum total cholesterol, and LDL cholesterol levels; as well as the the median values of plasma glucose level, serum insulin, triglyceride, AST, ALT, CRP levels, homeostasis assessment model of insulin resistance (HOMA-IR); and changes in LDL cholesterol level per year were significantly higher in the NAFLD group than in the normal group. The mean value of the HDL cholesterol level was significantly lower in the NAFLD group. The prevalence of obesity and diabetes mellitus and the proportion of monthly income less than 1 million Korean Won were higher in the NAFLD group. The proportion of individuals at moderate risk, high risk, and very high risk for CVD were significantly higher in the NAFLD group than the normal group.

### 3.2. Proportion of Non-Achievement of LDL Cholesterol Targets According to NAFLD Status Considering the Effect of Time

Table 2 shows the prediction of time effects on the proportion of non-achievement of LDL cholesterol targets according to NAFLD status using GEE models. In both overall and post-hoc analysis, the estimated proportions of non-achievement of LDL cholesterol targets in the NAFLD group remained higher than those in the normal group during all follow-up periods. The group × time interactions were also statistically significant.

### 3.3. Longitudinal Association between NAFLD and LDL-Cholesterol Target Out Events

**Figure 2:**
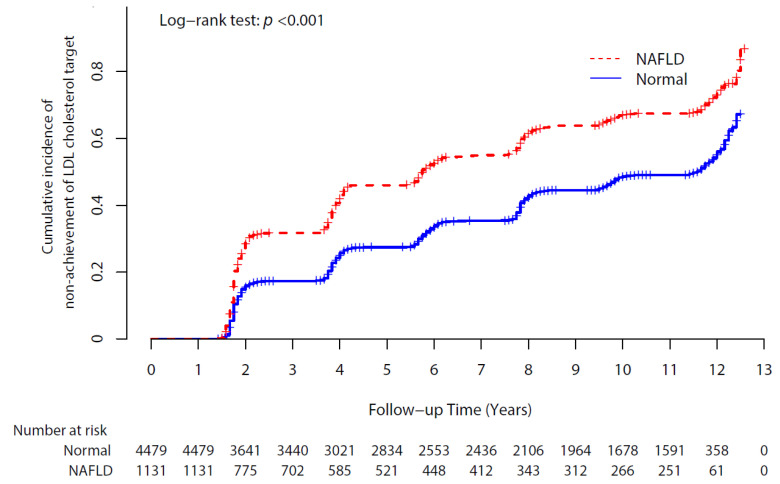
Cumulative incidence of non-achievement of LDL cholesterol targets according to NAFLD status. Abbreviations: LDL, low-density lipoprotein; NAFLD, non-alcoholic fatty liver disease.

A total of 2819 (50.3%) participants experienced non-achievement of their LDL cholesterol targets. The cumulative incidences of non-achievement of LDL cholesterol targets according to NAFLD status are presented in Figure 2 as Kaplan–Meier curves. The NAFLD group had higher cumulative incident non-achievement of LDL cholesterol targets than the normal group over 12 years of follow-up with significance (log-rank test *p* < 0.001).

Table 3 shows the independent relationships between NAFLD status and non-achievement of LDL cholesterol targets over a 12-year follow-up period. The HR with a 95% CI for non-achievement of LDL cholesterol targets in the NAFLD group, compared to the normal group, was 1.718 (1.578–1.870, *p* < 0.001). Similarly, a longitudinal association was noted after additional adjustment for age, sex, BMI, regular exercise, current smoker status, current drinker status, MBP, daily caloric intake, plasma glucose level, serum total cholesterol level, serum ALT level, serum CRP level, chronic diseases, treatment with anti-dyslipidemic medications, and changes in LDL cholesterol level per year. The corresponding adjusted HR with a 95% CI was 1.196 (1.057–1.353, *p =* 0.005).

**Figure 3:**
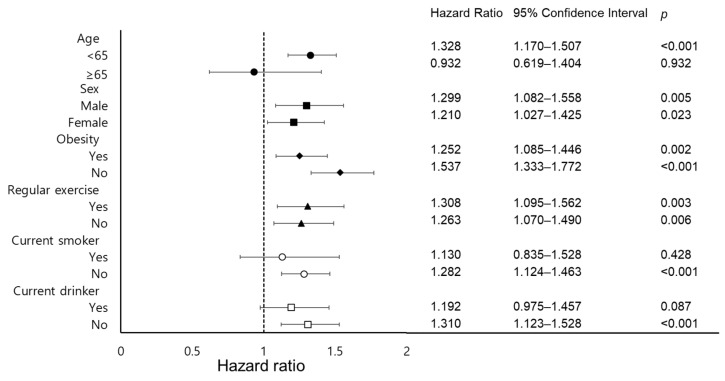
Subgroup analysis for associations between NAFLD and non-achievement of LDL targets. Abbreviations: NAFLD, non-alcoholic fatty liver disease; LDL, low-density lipoprotein; BMI, body mass index; ALT, alanine aminotransferase; CRP, C-reactive protein. Results were adjusted for age, sex, BMI, regular exercise, current smoker status, current drinker status, mean blood pressure, daily caloric intake, serum ALT level, serum CRP level, chronic diseases, treatment with anti-dyslipidemic medications, and changes in LDL cholesterol level per year except for each subgroup variable.

Figure 3 shows the results of subgroup analyses (age group, sex, obesity status, physical activity, smoking status, and alcohol drinking status) of the relationship between NAFLD status and non-achievement of LDL cholesterol targets, presented as HRs with 95% CI values. After adjusting for all potential confounding variables, except for each subgroup variable, there were significant associations between NAFLD and incident non-achievement of LDL cholesterol targets in the subgroup of individuals younger than 65 years in both sex subgroups, in both non-obesity and obesity subgroups, in both non-regular exercise and regular exercise subgroups, in non-smokers, and in non-drinkers, whereas older age, current smokers, and current drinkers did not show a significant relationship.

## 4. Discussion

We found that patients with NAFLD experienced unfavorable management of LDL cholesterol using data collected from a large-sample prospective cohort study in Korea.

In NAFLD, dyslipidemia is characterized by elevated triglyceride and LDL cholesterol and decreased HDL cholesterol [19]. Atherogenic dyslipidemia could increase the CVD risk in NAFLD patients. Previous studies have noted that NAFLD itself is a novel and independent risk factor of CVD. In a large multi-ethnic cohort, NAFLD was shown to be associated with higher triglyceride and LDL particle concentrations and lower LDL particle size and HDL cholesterol, independently of insulin resistance [20]. Hamaguchi et al. [21] showed that NAFLD, defined by ultrasonography, was a significant predictor of cardiovascular events after adjusting for conventional cardiovascular risk factors in Japanese men and women. In addition, Targher et al. [22] found that the risk of CVD events were significantly higher in diabetic individuals with NAFLD than in diabetics without NAFLD after adjusting for sex, age, smoking, diabetes duration, hemoglobin A1c, LDL cholesterol, medications, and metabolic syndrome (odds ratios: 1.87 (95% CIs: 1.20–2.60), *p* < 0.001). These results suggest that NAFLD increases the CVD risk independently of the presence of conventional risk factors (e.g., age, diabetes mellitus, and metabolic syndrome).

Several meta-analyses have sought to reveal associations between NAFLD, CVD risk, and mortality, although results have been inconsistent [23,24,25]. Targher et al. [23] found that NAFLD is significantly related with an increased risk of fatal and non-fatal cardiovascular events. Wu et al. [24] showed that NAFLD is associated with an increased risk of major adverse cardiovascular events, but not with all-cause mortality or CVD mortality. Another meta-analysis [25] found that NAFLD is associated with increased all-cause mortality, but not with CVD mortality or cancer mortality.

We found that fewer patients with NAFLD achieved their LDL cholesterol targets than individuals without NAFLD after considering possible confounders. In the subgroup analysis, similar results were shown regardless of sex, obesity, and regular exercise. Although the estimated proportion of individuals that did not achieve their LDL cholesterol targets would vary with time, this proportion was higher in the NAFLD group than in the normal group throughout the entire 12-year follow-up period. Indeed, the difference in this proportion between groups decreased with time (group × time interaction *p* = 0.0005). Even in the normal group without NAFLD at the baseline survey, metabolic diseases such as NAFLD, metabolic syndrome, and diabetes mellitus could occur in the group over time, which could lead to a decrease in the difference of the estimated proportion of participants with non-achievement of LDL cholesterol targets between the groups. Time-dependent Cox proportional regression analysis should be performed considering the occurrence of NAFLD over time in future research. In subgroup analysis, similar results were observed regardless of sex, obesity, and regular exercise, which suggests that no interaction was present in those variables.

Interestingly, a population-based study in the United States reported that the use of lipid-lowering drugs, including statins, was not associated with overall and CVD mortality in patients with NAFLD [26]. The authors suggested that the underuse of anti-dyslipidemic drugs in NAFLD patients with dyslipidemia might be related to this result. They also suggested that other CVD risk factors, including diabetes and smoking, could have a greater impact on CVD mortality in NAFLD patients. Our findings could support this previous report. Altogether, we showed that NAFLD patients did not reach their optimal LDL management target levels after adjusting for the use of anti-dyslipidemia medication. This means that NAFLD might confer an excess risk above underlying metabolic risk factors. However, these findings are in contrast with the results of three post hoc analyses of randomized controlled trials [27]. The data from the post hoc analyses suggested that statin treatment reduced CVD morbidity and mortality in NAFLD/NASH patients. Thus, the effect of lipid-lowering drugs on mortality in NAFLD patients remains to be established. Other pathogenic abnormalities that increase mortality in NAFLD patients should be considered in future studies.

There is the possibility of information bias in our study because the proportion of participants who responded that they had taken anti-dyslipidemic medications was strikingly low (0.3%) at the baseline survey, and 11.7% of the participants responded that they had taken anti-dyslipidemic medications during the follow-up period. Follow-up studies should be performed considering time-varying proportions of participants with anti-dyslipidemic medications to verify the effect of anti-dyslipidemic medications in NAFLD patients. In addition, there is a lack of information about the specific type of anti-dyslipidemic medications in the KoGES data. In the baseline survey from 2001 to 2002, it is possible that respondents were relatively unaware of whether they were taking anti-dyslipidemic medications. Although lipid-lowering treatment should be intensified, especially in groups with a high CVD risk and with a very high CVD risk according to current guidelines [7,28], prior guidelines on the management of dyslipidemia, which set the LDL cholesterol level goal below 100 mg/dL in coronary heart disease (CHD), and the equivalent CHD risk assessment made doctors prescribe anti-dyslipidemic medications less than they do now [29].

A few possible mechanisms may underlie our results. The first is insulin resistance induced by NAFLD. NAFLD occurs when the amount of triglyceride synthesis in the liver exceeds the amount of triglyceride expenditure [30], and de novo lipogenesis increases [31]. In NAFLD, hepatic glucose production and insulin sensitivity are impaired [32]. Insulin resistance contributes to an increase in circulating LDL cholesterol levels by upregulating hepatic lipase and protein convertase subtilisin/kexin type 9 (PCSK9) [33,34]. Second, hepatic steatosis damages cell-surface LDLR by inducing de novo PCSK9 expression in mice [35]. The expression of sterol response element binding protein-1c (SREBP-1c), a major regulator of fatty acid synthesis, has been shown to be higher in NAFLD patients [36]. LDL receptor (LDLR) gene expression has been found to be lower in patients with NAFLD compared to those without NAFLD [37]. Furthermore, research has indicated that LDLR gene expression does not differ in NAFLD patients on or off statins [38]. Third, excessive production of free fatty acids could also be an important factor influencing both the development of NAFLD and non-achievement of LDL cholesterol management. In the liver, the activation of transcription factors, such as carbohydrate responsive element binding protein (ChREBP) and SREBP-1c, helps to synthesize fatty acids in the liver by interacting with the delivery of chylomicron cholesterol from the intestinal lumen, which contributes to NAFLD [39]. A portion of chylomicron is incorporated into very-low-density lipoprotein particles, the source of plasma LDL cholesterol, which may act as a factor that interferes with the management of LDL cholesterol [40]. Fourth, NAFLD has been shown to be associated with increased SREBP-2 maturation, 3-hydroxy-3-methylglutaryl coenzyme A (HMG CoA) reductase expression, and decreased phosphorylation of HMG CoA reductase [38]. Altogether, these results suggest that disrupted cholesterol metabolism in NAFLD may lead to increased CVD risk.

In this study, the association between NAFLD and the non-achievement of LDL cholesterol targets disappeared in subgroups of individuals older than 65 years, current smokers, and current drinkers. These factors (aging, smoking, and alcohol drinking) might act as more powerful confounders in the management of LDL cholesterol than NAFLD.

This study has several limitations. First, participants were diagnosed with NAFLD using a biomarker-based prediction model, the NAFLD liver fat score, rather than using imaging tests or histologic findings. Therefore, we investigated the association between NAFLD using another surrogate marker (hepatic steatosis index, HSI) and non-achievement of LDL cholesterol targets. Although it was not statistically significant, the non-achievement of LDL cholesterol target tended to be associated with NAFLD status using HSI (Tables S1 and S2). Further studies are needed to analyze the management of LDL cholesterol according to the severity of fatty liver disease based on imaging tests or liver biopsy. Second, we could not exclude causes of secondary fatty liver, including autoimmune hepatitis, Wilson’s disease, and medication-induced fatty liver, due to the lack of data. To overcome this limitation, however, we excluded heavy alcoholics as well as individuals who had a history of hepatitis infection, hepatitis B viral infection, or hepatitis C viral infection, which account for a major proportion of chronic liver diseases in Korea [41]. Third, we could not assess the potential effect of changes in hepatic steatosis over time on the association between NAFLD and CVD risk. In the next study, we will further analyze the association between the non-achievement of LDL cholesterol targets and NAFLD regarding the changes in NAFLD status (regression group, transient remission group, and persistent group), as in the previous study [42]. Fourth, we assessed CVD risk levels by applying Korean guidelines and thus our results may not be applicable to other countries. In addition, the lack of information on carotid artery stenosis, peripheral artery disease, and abdominal aortic aneurysm in the KoGES may have led to the underestimation of major CVD risk factors among the participants. To the best of our knowledge, however, this is the first study to report on the longitudinal effect of NAFLD on the management of LDL cholesterol levels in the consideration of an individual’s overall CVD risk. In addition, this relationship remained significant independently of sex and obesity.

## 5. Conclusions

We found that NAFLD was significantly associated with non-achievement of LDL targets in a population-based prospective study. Our results suggest that prevention and proper management of NAFLD have important health implications for the prevention of CVD.

## Figures and Tables

**Table 1:** Baseline characteristics of the study population.

	KoGES: Ansan_Ansung Study		
	Normal	NAFLD	*p*
Numbers (n)	4479	1131	
Male, *n* (%)	1716 (38.3)	495 (43.8)	0.001
Age, years	51.0 ± 8.8	52.9 ± 8.6	<0.001
Waist circumference, cm	79.6 ± 8.2	88.8 ± 7.8	<0.001
BMI, kg/m^2^	23.8 ± 2.8	26.5 ± 3.0	<0.001
Obesity, *n* (%)	1124 (25.1)	591 (52.3)	<0.001
Mean blood pressure, mmHg	90.4 ± 12.3	99.7 ± 12.1	<0.001
Glucose, mg/dL	81 (76, 85)	84 (78, 92)	<0.001
Insulin, µU/mL	6.4 (4.9, 8.4)	10.4 (8.1, 13.0)	<0.001
HOMA-IR	1.3 (1.0, 1.7))	2.2 (1.7, 2.8)	<0.001
Total cholesterol, mg/dL	178.9 ± 27.6	187.6 ± 32.4	<0.001
Triglyceride, mg/dL	116 (90, 154)	201 (152, 283)	<0.001
HDL cholesterol, mg/dL	46.0 ± 10.0	39.3 ± 8.6	<0.001
LDL cholesterol, mg/dL	103.1 ± 26.2	106.4 ± 24.1	<0.001
Changes in LDL cholesterol level, mg/dL/year	3.0 (0.6, 8.4)	4.7 (0.9, 15.0)	<0.001
AST, IU/L	25 (22, 29)	29 (24, 36)	<0.001
ALT, IU/L	20 (16, 26)	31 (22, 45)	<0.001
CRP, mg/dL	0.1 (0.1, 0.2)	0.2 (0.1, 0.3)	<0.001
Monthly household income, *n* (%)			<0.001
<1 million Korean Won	1476 (33.6)	446 (40.6)	<0.001
1–2 million Korean Won	1400 (31.8)	295 (26.9)	0.001
>2 million Korean Won	1522 (34.6)	357 (32.5)	0.191
Employment status, *n* (%)	2432 (54.7)	631 (56.4)	0.314
Regular exercise, *n* (%)	2223 (51.3)	529 (48.5)	0.101
Current smoker, *n* (%)	832 (18.9)	236 (21.2)	0.074
Current drinker, *n* (%)	1917 (43.2)	449 (40.1)	0.062
Daily amount of alcohol intake, g/day	5 (2, 12)	8 (3, 18)	<0.001
Daily caloric intake, kcal/day	1820 (1500, 2198)	1860 (1534, 2291)	0.022
Daily carbohydrate intake, g/day	322 (275, 382)	333 (284, 405)	<0.001
Daily protein intake, g/day	61 (47, 77)	61 (48, 78)	0.554
Daily fat intake, g/day	28 (19, 40)	27 (17, 39)	0.016
Chronic diseases, *n* (%) *	136 (3.0)	34 (3.0)	0.960
Diabetes mellitus, *n* (%)	22 (0.5)	105 (9.3)	<0.001
CVD risk group, *n* (%)			<0.001
Low risk group	3113 (69.5)	440 (38.9)	<0.001
Moderate risk group	1337 (29.9)	582 (51.5)	<0.001
High risk group	16 (0.4)	34 (3.0)	<0.001
Very high risk group	13 (0.3)	75 (6.6)	<0.001

Abbreviations: NAFLD, non-alcoholic fatty liver disease; BMI, body mass index; HOMA-IR, homeostasis assessment model of insulin resistance; HDL, high-density lipoprotein; LDL, low-density lipoprotein; AST, aspartate aminotransferase; ALT, alanine aminotransferase; CRP, C-reactive protein; CVD, cardiovascular disease. * Chronic disease was defined as having at least one of the following: chronic obstructive pulmonary disease or any chronic cancers. Data are presented as means ± standard deviations or medians (interquartile range). *p* was calculated using the independent *t*-test or Mann–Whitney U-test for continuous variables and the Chi-squared test for categorical variables.

**Table 2:** Generalized estimating equation models predicting the effects of time on the proportion of non-achievement of LDL cholesterol targets according to NAFLD status.

	Normal		NAFLD			
Time	Estimated Proportion (%)	SE	Estimated Proportion (%)	SE	Overall *p*	Post-Hoc *p*
1st f/u	17.91	0.588	32.32	1.421	group: <0.001 time: <0.001 group × time: 0.0005	<0.0001
2nd f/u	18.77	0.637	30.54	1.500		<0.0001
3rd f/u	22.13	0.715	33.60	1.605		<0.0001
4th f/u	28.03	0.770	41.41	1.702		<0.0001
5th f/u	21.21	0.722	27.72	1.593		0.0002
6th f/u	22.27	0.749	28.53	1.657		0.0006

Abbreviations: LDL, low-density lipoprotein; NAFLD, non-alcoholic fatty liver disease; SE, standard error. group×time means interactions between group and time.

**Table 3:** Relationship between NAFLD status and non-achievement of LDL cholesterol targets.

	Unadjusted		Adjusted *	
	HR (95% CI)	*p*	HR (95% CI)	*p*
NAFLD (vs. Normal)	1.718 (0.578–1.870)	<0.001	1.196 (1.057–1.353)	0.005
Male (vs. Female)	1.007 (0.933–1.086)	0.866	1.253 (1.114–1.410)	<0.001
Age (per 1 increment)	1.020 (1.016–1.024)	<0.001	1.020 (1.015–1.026)	<0.001
BMI (per 1 increment)	1.072 (1.058–1.086)	<0.001	1.030 (1.014–1.047)	<0.001
Regular exercise (vs. non-regular exercise)	0.957 (0.888–1.031)	0.247	0.931 (0.852–1.019)	0.120
Current smoker (vs. ex-/non-smoker)	1.132 (1.031–1.243)	0.010	1.412 (1.231–1.620)	<0.001
Current drinker (vs. non-drinker)	1.124 (1.042–1.212)	0.003	1.262 (1.139–1.398)	<0.001
Mean blood pressure (per 1 increment)	1.014 (1.012–1.017)	<0.001	1.007 (1.003–1.011)	<0.001
Daily caloric intake (per 1 increment)	1.000 (1.000–1.000)	0.113	1.000 (1.000–1.000)	0.361
Glucose (per 1 increment)	1.008 (1.007–1.010)	<0.001	1.010 (1.005–1.015)	<0.001
Total cholesterol (per 1 increment)	1.017 (1.016–1.018)	<0.001	1.019 (1.017–1.021)	<0.001
ALT (per 1 increment)	1.002 (1.001–1.002)	<0.001	1.000 (0.999–1.001)	0.718
CRP (per 1 increment)	1.056 (0.996–1.120)	0.069	1.137 (1.006–1.284)	0.040
Chronic diseases (vs. without chronic diseases)	1.071 (0.871–1.317)	0.514	1.043 (0.814–1.336)	0.741
Anti-dyslipidemic medications (vs. without anti-dyslipidemic medications)	2.061 (1.140–3.725)	0.017	0.358 (0.155–0.823)	0.016
Changes in LDL cholesterol level per year (per 1 increment)	1.089 (1.086–1.092)	<0.001	1.087 (1.083–1.091)	<0.001

Abbreviations: NAFLD, non-alcoholic fatty liver disease; LDL, low-density lipoprotein; HR, hazard ratio; CI, confidence interval; BMI, body mass index; ALT, alanine aminotransferase; CRP, C-reactive protein. * Adjusted for age, sex, BMI, regular exercise, current smoker status, current drinker status, mean blood pressure, daily caloric intake, plasma glucose level, serum total cholesterol level, serum ALT level, serum CRP level, chronic diseases, treatment with anti-dyslipidemic medications, and changes in LDL cholesterol level per year.

## Data Availability

The data that support the findings of this study are available from the Korea Centers for Disease Control and Prevention (KCDC). Restrictions apply to the availability of these data, which were used under license for this study. Data are available at http://www.cdc.go.kr with the permission of KCDC (Accessed date: 20 February 2021).

## Supplementary Materials

The following are available online at https://www.mdpi.com/article/10.3390/ijerph18073442/s1, Table S1: Relationship between hepatic steatosis index and non-achievement of LDL cholesterol targets, Table S2: Relationship between NAFLD status using hepatic steatosis index and non-achievement of LDL cholesterol targets.
