# Supplementary material for: Non-Alcoholic Fatty Liver Disease Is an Independent Risk Factor for LDL Cholesterol Target Level
Source: Int J Environ Res Public Health. 2021 Mar 26;18(7):3442. doi: 10.3390/ijerph18073442 (PMC8037151; doi:10.3390/ijerph18073442)
Supplement: Supplementary file 1 [file ijerph-18-03442-s001.pdf]

Table S1. Relationship between HSI and non-achievement of LDL cholesterol target.

	Unadjusted		Adjusted*	
	HR (95% CI)	*p*	HR (95% CI)	*p*
HSI (per 1 increment)	1.057 (1.047–1.067)	<0.001	1.101 (0.984–1.231)	0.094

*: Adjusted for age, sex, BMI, regular exercise, current smoker, current drinker, mean blood pressure, daily caloric intake, serum ALT level, serum CRP level, chronic diseases, treatment with anti-dyslipidemic medications, and changes in LDL cholesterol level per year.

Abbreviations: HSI, hepatic steatosis index; LDL, low-density lipoprotein; HR, hazard ratio; CI, confidence interval; BMI, body mass index; ALT, alanine aminotransferase; CRP, C-reactive protein.

Table S2. Relationship between NAFLD status using HSI and non-achievement of LDL cholesterol target

	Fatty liver ruled out	Fatty liver neither ruled in nor ruled out		Fatty liver ruled in	
		HRs (95% CIs)	*p*	HRs (95% CIs)	*p*
Unadjusted	1 (reference)	1.418 (1.276–1.576)	<0.001	1.887 (1.670–2.132)	<0.001
Model 1	1 (reference)	1.327 (1.158–1.521)	<0.001	1.534 (1.243–1.894)	<0.001
Model 2	1 (reference)	1.176 (1.025–1.350)	0.021	1.120 (0.900–1.393)	0.302
Model 3	1 (reference)	1.134 (0.982–1.308)	0.086	1.209 (0.965–1.514)	0.099

Model 1: Adjusted for age, sex, BMI, regular exercise, current smoker, current drinker, mean blood pressure, and daily caloric intake.

Model 2: Adjusted for variables in Model 1 plus serum ALT level, serum CRP level, and chronic diseases.

Model 3: Adjusted for variables in Model 2 plus treatment with anti-dyslipidemic medications, and changes in LDL cholesterol level per year.

Abbreviations: NAFLD, non-alcoholic fatty liver disease; HSI, hepatic steatosis index; LDL, low-density lipoprotein; HRs, hazard ratios; CIs, confidence intervals; BMI, body mass index; ALT, alanine aminotransferase; CRP, C-reactive protein.
